# Appetite, coping strategies, and morale in older adults with advanced gastrointestinal cancer: a longitudinal observational study

**DOI:** 10.1186/s12877-026-07532-5

**Published:** 2026-04-21

**Authors:** Kaori Yagasaki, Tomoko Arahata, Noriyuki Ishida, Kenro Hirata, Mai Funato, Yasuo Hamamoto

**Affiliations:** 1 Faculty of Nursing and Medical Care, Keio University, 35 Shinanomachi, Shinjuku, Tokyo 160-8582 Japan; 2 Center for Development of Advanced Cancer Therapy, Cancer Institute Hospital of Japanese Foundation for Cancer Research, 3-8-31, Ariake, Koto, Tokyo 135-8550 Japan; 3 Cancer Center, Keio University School of Medicine, 35 Shinanomachi, Shinjuku, Tokyo 160-8582 Japan; 4 Department of Medical Oncology, Institute of Science Tokyo , 1-5-45, Yushima, Bunkyo-ku, Tokyo 113-8510 Japan

**Keywords:** Appetite change, Coping strategies, Morale, Gastrointestinal cancer, Cancer, Longitudinal study, Observational study

## Abstract

**Background:**

Older adults with advanced gastrointestinal cancer face diverse physical challenges associated with the disease, treatments, and aging. Maintaining morale is crucial for these patients, as it enables them to sustain psychological well-being and a sense of fulfillment. We aimed to assess the relationships between appetite, coping strategies, and changes in morale after three months of follow-up in older adults with advanced gastrointestinal cancer.

**Methods:**

A three-month longitudinal observational study was conducted among 79 older adults (aged 70 and over) with advanced gastrointestinal cancer receiving chemotherapy as outpatients at a hospital in Japan between 2022 and 2024. We assessed morale using the Philadelphia Geriatric Center Morale Scale, appetite using the Simplified Nutritional Appetite Questionnaire, coping strategies using the Brief Coping Orientation to Problems Experienced Inventory, frailty using the Geriatric 8 scale, and symptoms using the MD Anderson Symptom Inventory. A linear mixed-effects model was used to estimate the difference in change in morale from baseline to three months later between high- and low-appetite groups.

**Results:**

Data from 66 participants who completed the follow-up were analyzed. The participants (mean age = 76.8 years) had a mean nutritional appetite score of 14.3, morale score of 12.0, and frailty score of 11.7. Acceptance was the most frequently used coping strategy. At three months from baseline, morale was lower in the low-appetite group than in the high-appetite group. While the change in morale over three months did not differ significantly between the two appetite groups, the use of substances as a coping strategy was significantly associated with lower morale after three months (*p* = 0.01).

**Conclusions:**

While low baseline appetite was associated with lower morale, it was not significantly associated with changes in morale over three months. Instead, reliance on substance use as a coping strategy appeared more important. Therefore, monitoring substance use may be useful for identifying patients who need greater psychological support. Additionally, considering support methods that enable older adults to enjoy eating aligned with their values might be beneficial. These results serve as a foundation for future research aimed at understanding and supporting the morale of older adults with gastrointestinal cancer.

**Supplementary Information:**

The online version contains supplementary material available at 10.1186/s12877-026-07532-5.

## Background

Gastrointestinal cancer accounts for a quarter of global cancer incidence and one-third of cancer-related deaths [1], with incidence and mortality increasing significantly with age [2]. Recent advances in early detection, combination therapy, and immunotherapy have provided treatment options with less toxicity and more opportunities for treatment tolerance in older adults with cancer, which has increased survival rates [3]. However, palliative chemotherapy is often associated with malnutrition, which poses a significant challenge to maintaining patients’ quality of life (QOL) [4].　Nutritional risks are common in older adults with gastrointestinal cancer [5], particularly those undergoing chemotherapy. Eating difficulties and nutrition loss contribute to physical frailty, and these factors are linked to survival and QOL [4]. Moreover, eating problems among adults with gastrointestinal cancer have a socio-emotional impact because of decreased enjoyment of food [6]. For instance, chemotherapy-induced changes in smell and taste can affect flavor perception, negatively influencing food preferences and eating habits [7].

Understanding the coping strategies employed by older adults with gastrointestinal cancer is crucial for managing these eating-related challenges. Coping strategies refer to the psychological efforts made to deal with stressful events [8]. A study showed that despite suffering daily from the impact of cancer and its treatment, older adults with advanced gastrointestinal cancer proactively solved problems and reassessed their position to “eat to live” [9]. These older adults rediscovered the pleasure of eating and actively used coping strategies to deal with dietary issues.

Such coping resources can be developed throughout the life course and strengthened even in old age [10]. Closely linked to this adaptive capacity is the concept of morale. Sullivan et al. [11] highlight the importance of morale for improving the lives of older adults with chronic diseases. Morale is a multidimensional concept related to social, functional, and medical factors that is often synonymous with subjective or psychological well-being, QOL, and life satisfaction [12, 13]. Crucially, Niklasson et al. [14] identify high morale as a salutogenic factor. For older adults with advanced gastrointestinal cancer, focusing on morale is essential; it enables them to preserve psychological health and maintain a sense of fulfillment, even in the face of physical decline. While previous studies have examined morale in general older adults [14, 15], research on cancer and morale is limited. Essentially, coping and morale are dynamic processes, particularly for older adults facing the evolving challenges of continuous chemotherapy such as worsening appetite and symptom exacerbation. Assessment at a single time point is insufficient to capture these adaptations. Consequently, the longitudinal relationships between eating problems, coping strategies, and morale remain unclear in this population. Thus, we aimed to assess the longitudinal relationships between appetite, coping strategies, and morale after three months of follow-up among older adults with advanced gastrointestinal cancer.

## Methods

### Study design

This longitudinal observational study was conducted at three time points: baseline (T0) after regimen change, one month later (T1), and three months later (T2). This duration was selected to ensure feasibility and minimize loss to follow-up due to worsening health conditions in older adults with advanced gastrointestinal cancer.

### Participants and setting

Participants were recruited between August 16, 2022 and March 21, 2024 from the gastrointestinal oncology outpatient department or oncology center of a university hospital in Tokyo. Inclusion criteria were: 1) age ≥ 70 years; 2) diagnosis of gastrointestinal cancer (stomach, colorectal, liver, biliary [gallbladder/bile duct], or pancreatic); and 3) undergoing the first cycle of a new regimen. Exclusion criteria included esophageal cancer or gastrointestinal stromal tumors, significant physical or mental burden, and inability to read or write Japanese.

Those who met the eligibility criteria and provided informed consent were enrolled in the study. The sample size was determined based on feasibility, considering the available patient population, previous studies in this field, and consultation with a biostatistician, rather than a formal power analysis.

Two researchers (KY and TA) identified eligible candidates, and YH and KH introduced the study during the patients’ visits to the gastrointestinal oncology outpatient clinic or oncology center.

### Ethical approval

This study (clinical trial number: UMIN000048556||http://www.umin.ac.jp/ctr/; August 15, 2022) was approved by the Institutional Review Boards of Keio University School of Medicine (No. 20221058) and Nursing and Medical Care, Keio University (No. 202302). All participants provided written informed consent.

### Data collection

The baseline (T0) survey was conducted in the outpatient department using a self-administered questionnaire. T1 and T2 follow-up surveys were conducted via mail or during outpatient visits. The demographic and clinical data (e.g., medications, hemoglobin, serum albumin, C-reactive protein, and Eastern Cooperative Oncology Group performance status) were obtained from electronic medical records.

### Measures

The measures and measurement times used in this study are shown in Table 1.

**Table 1:** Measures and measurement times

Measures	Measurement time		
	T0	T1	T2
Demographic and medical information (from electronic medical records)	✓		
Revised Philadelphia Geriatric Center Morale Scale	✓	✓	✓
Simplified Nutritional Appetite Questionnaire	✓	✓	✓
Brief Coping Orientation to Problems Experienced Inventory	✓		
MD Anderson Symptom Inventory-Japanese Version	✓	✓	✓
Geriatric 8 scale	✓		

*Abbreviations*: *T0* after new regimen change, *T1* after one month, *T2* after three months

### Revised Philadelphia geriatric center morale scale

Morale was measured using the 17-item Revised Philadelphia Geriatric Center Morale Scale (PGCMS-Re) [13]. The original PGCMS is a 22-item measure of morale, psychological well-being, and QOL in older populations in communities and institutions [16]. It consists of three domains: Attitude Toward Own Aging (five items), Agitation (six items), and Lonely Dissatisfaction (six items). Each item uses a two-choice response format, with total scores ranging from 0 to 17. Higher scores indicate higher morale. Scores of 13–17, 10–12, and ≤ 9 indicate high, moderate, and low levels of morale, respectively [17]. The validity and reliability of the Japanese version have been verified [18, 19], with a Kuder–Richardson 20 coefficient of 0.85–0.86 [18].

### Simplified nutritional appetite questionnaire

Appetite was assessed using the Japanese version of the Simplified Nutritional Appetite Questionnaire (SNAQ) [20], comprising four items evaluated on a 5-point scale. Total scores range from 4 to 20; higher scores indicate a better appetite. Scores ≤ 14 indicate a significant risk of at least 5% weight loss within six months [21]. In this study, baseline SNAQ scores ≤ 14 were classified as low appetite and those ≥ 15 as high appetite. Cronbach’s alpha was 0.55 (considered acceptable for short scales) and the test–retest reliability was good (intraclass correlation coefficient = 0.75) [20], supporting the scale’s stability.

### Brief coping orientation to problems experienced inventory

The Brief Coping Orientation to Problems Experienced Inventory (Brief COPE) [22] is a shortened version of the COPE [23]. Coping strategies were measured using the Japanese version of the Brief COPE [24]. It is a comprehensive 28-item self-report questionnaire with 14 subscales (e.g., active coping, acceptance, substance use, use of emotional support). Responses are rated on a 4-point scale ranging from 1 (“I haven’t been doing this at all”) to 4 (“I’ve been doing this a lot”). Notably, the “substance use” subscale encompasses the use of alcohol and medications for non-medical purposes. The score for each subscale ranges from 2 to 8, with higher scores indicating greater use of that strategy [25]. The reliability and validity of the Japanese version have been verified, with Cronbach’s alpha values ranging from 0.46 to 0.96 [24].

### MD Anderson Symptom Inventory-Japanese version

Symptom burden was evaluated using the MD Anderson Symptom Inventory-Japanese Version (MDASI-J) [26], which is a self-administered questionnaire for evaluating the severity of symptoms experienced by patients with cancer and the consequent interference with daily living [26]. It includes 13 symptom items (e.g., pain, fatigue, nausea), and six interference items (e.g., activities, mood, work [including housework]). The reliability and validity of the Japanese version are established, with Cronbach’s alpha coefficients of 0.92 for symptoms and 0.93 for interference [27].

### Geriatric 8 scale

Frailty was assessed using the Japanese version of the Geriatric 8 scale (G-8), a comprehensive screening tool for older adults [28]. It comprises eight items (of which seven are related to physical function, medication, nutrition, cognition, and mood) from the Mini Nutritional Assessment [29], a tool for assessing nutritional status among older adults. Total scores range from 0 to 17; higher scores indicate better health, whereas scores of ≤ 14 are classified as abnormal. While internal consistency reports for the Japanese version are limited, its validity for functional status screening in older patients with cancer has been demonstrated [30].

### Data analysis

Descriptive statistics were calculated for all eligible participants and the low- and high-appetite groups. Participant characteristics were summarized as percentages for categorical variables and means with standard deviations for continuous variables. The 14 coping strategy subscales were similarly summarized and compared across groups, with frequencies and percentages for scores of 2–5 and 6–8 at baseline. Regarding the borderline score of 5, while our data confirmed that all participants with this score answered in the “2 (A little bit) + 3 (A medium amount)” pattern, their average score was 2.5; as this fell below the threshold of “a medium amount” (3.0), we categorized participants with a score of 5 into the lower utilization group.

A linear mixed-effects model was used to estimate the difference in change in morale from baseline to T2 between the two groups. The fixed effects included group, period, and the interaction between group and period. Additionally, age and sex, metastasis, and baseline morale scores were included as covariates to adjust for demographic characteristics, disease severity, and initial differences, respectively. Random effects were included to account for the repeated measures within participants. The degrees of freedom were calculated using the Kenward–Roger method, and an unstructured covariance structure, determined according to the Akaike information criterion, was employed.

All statistical analyses were performed using SAS version 9.4 (SAS Institute, Inc, Cary, NC, USA). All *p*-values were two-sided, and *p* < 0.05 was considered statistically significant.

## Results

### Participant characteristics and relationships among variables

**Fig. 1:**
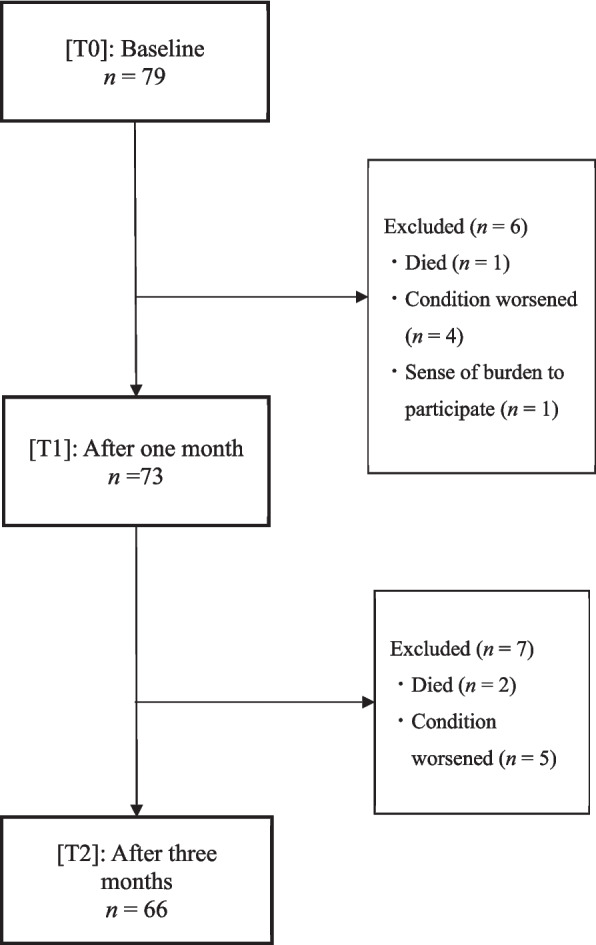
Flowchart of participant retention

Of the 79 older adult outpatients with gastrointestinal cancer included initially, 13 dropped out during the three-month follow-up period and four participants with missing T1/T2 data and one with missing baseline SNAQ data were excluded. This resulted in a final analytic sample of 65 participants (Fig. 1).

Table 2 shows the participants’ characteristics. The analysis revealed that, compared with the high-appetite group (SNAQ ≥ 15), the low-appetite group (SNAQ ≤ 14) exhibited significantly greater symptom severity (*p* = 0.002), impact on daily life (*p* = 0.0007), functional impairment (lower G-8 scores), and analgesic use (*p* = 0.01). The mean PGCMS-Re score (12.0, standard deviation = 3.0) was classified as moderate. In addition, the low-appetite group had lower morale compared with the high- appetite group (*p* = 0.0004). In other words, at baseline, symptom severity was associated with appetite, and appetite levels were associated with morale.

**Table 2:** Participants’ baseline demographic and clinical characteristics

Variables	Total (*n* = 66)	Low-appetite group (*n* = 34)	High-appetite group (*n* = 31)
Sex, *n* (%)			
Male	35 (53.0)	18 (52.9)	17 (54.8)
Female	31 (47.0)	16 (47.1)	14 (45.2)
Age (years), mean (SD)	76.8 (4.4)	76.6 (4.7)	77.1 (4.1)
BMI, mean (SD)	21.4 (3.6)	21.3 (3.6)	21.6 (3.8)
CRP (mg/dl), mean (SD), *n* = 65	0.9 (1.5)	1.0 (1.5)	0.8 (1.5)
ALB (g/dl), mean (SD)	3.6 (0.5)	3.7 (0.4)	3.6 (0.6)
Hemoglobin (g/dl), mean (SD)	11.1 (1.7)	11.4 (1.6)	10.6 (1.8)
Marital status, *n* (%)			
Married	59 (93.7)	29 (93.5)	29 (93.5)
Unmarried	4 (6.3)	2 (6.5)	2 (6.5)
Missing data	3	3	0
Living circumstance, *n* (%)			
Living alone	13 (20.0)	8 (24.2)	5 (16.1)
Living together	52 (80.0)	25 (75.8)	26 (83.9)
Missing data	1	1	0
Alcohol history, *n* (%)			
No	41 (69.5)	23 (74.2)	17 (63.0)
Yes	18 (30.5)	8 (25.8)	10 (37.0)
Cancer site, *n* (%)			
Colorectal	26 (39.4)	13 (38.2)	12 (38.7)
Hepato-biliary-pancreatic	21 (31.8)	10 (29.4)	11 (35.5)
Gastric	13 (19.7)	7 (20.6)	6 (19.4)
Duodenum	4 (6.1)	2 (5.9)	2 (6.4)
Small intestine	2 (3.0)	2 (5.9)	0 (0.0)
Metastasis, *n* (%)			
Without metastasis	5 (7.6)	1 (2.9)	3 (9.7)
With metastasis	61 (92.4)	33 (97.1)	28 (90.3)
History of treatment, *n* (%)			
Surgery	49 (74.2)	22 (64.7)	26 (83.9)
Chemotherapy	33 (50.0)	16 (47.1)	17 (54.8)
Radiotherapy	0 (0.0)	0 (0.0)	0 (0.0)
ECOG performance status, *n* (%)			
0	30 (45.5)	14 (41.2)	16 (51.6)
1	34 (51.5)	18 (52.9)	15 (48.4)
2	1 (1.5)	1 (2.9)	0 (0.0)
3	1 (1.5)	1 (2.9)	0 (0.0)
4	0 (0.0)	0 (0.0)	0 (0.0)
Medication at present, *n* (%)			
NSAIDs・Acetaminophen	16 (24.2)	13 (38.2)	3 (9.7)
Opioids	3 (4.6)	3 (8.8)	0 (0.0)
Sleeping pills	5 (7.6)	1 (2.9)	4 (12.9)
Antiemetics	17 (25.8)	11 (32.4)	6 (19.4)
Anamorelin hydrochloride	4 (6.1)	2 (5.9)	2 (6.5)
Others	52 (78.9)	28 (82.4)	23 (74.2)
Stoma, *n* (%)			
With stoma	6 (9.1)	1 (2.9)	5 (16.1)
None	60 (90.9)	33 (97.1)	26 (83.9)
PGCMS-Re, mean (SD),* n* = 61	12.0 (3.0)	10.7 (3.0)	13.4 (2.3)
MDASI-J, mean (SD), *n* = 63			
Symptom items	1.7 (1.5)	2.3 (1.6)	1.1 (0.9)
Interference items	2.1 (1.9)	2.9 (2.1)	1.2 (1.1)
SNAQ, mean (SD), *n* = 65	14.3 (2.1)	12.0 (1.3)	16.0 (1.1)
G-8, mean (SD)	11.7 (2.5)	10.6 (2.4)	13.1 (2.0)
G-8, *n* (%)			
≦14	55 (83.3)	31 (91.2)	23 (74.2)
> 14	11 (16.7)	3 (8.8)	8 (25.8)

*Abbreviations*: *ALB* albumin, *BMI* body mass index, *CRP* C-reactive protein; small intestine, small intestine including the cecum, *NSAIDs* nonsteroidal anti-inflammatory drugs, *ECOG* Eastern Cooperative Oncology Group, *PGCMS-Re* 17-item Philadelphia Geriatric Center Morale Scale, *MDASI-J* MD Anderson Symptom Inventory-Japanese Version, *SNAQ* Simplified Nutritional Appetite Questionnaire, *G-8* Geriatric 8 scale

One participant had missing data for all SNAQ items at baseline, resulting in the analysis of 65 participants. Numbers are presented as *n* (%) or means (SD; standard deviation)

Furthermore, the non-completer (13 dropout) group exhibited significantly poorer nutritional and inflammatory status at baseline (Table 2 and Supplementary Materials 1) (e.g., a lower mean serum albumin value [3.3 vs. 3.6 for completers], a higher mean C-reactive protein level [3.2 vs. 0.9 for completers], and a lower mean SNAQ score [12.4 vs. 14.3 for completers]). Consistent with these markers, a significantly higher proportion of dropouts used nonsteroidal anti-inflammatory drugs (61.5% vs. 24.2%). While coping strategies did not differ significantly between groups, these findings suggest that dropouts represented a nutritionally more vulnerable subgroup, indicating potential for attrition bias (Supplementary Materials 1 and 2).

### Use of coping strategies

Table 3 shows participants’ coping strategies at baseline. Participants predominantly used adaptive and emotion-focused strategies, such as acceptance, positive reframing, and active coping, while maladaptive strategies (e.g., self-blame and denial) were rare. Regarding appetite, those with lower appetite (vs. higher appetite) showed a higher tendency to use emotional support (*p* = 0.07) and venting (*p* = 0.06).

**Table 3:** Participants’ coping strategies

Variables	Score level	Total (*n* = 65)	Low-appetite group (*n* = 34)	High-appetite group (*n* = 31)
		*n* (%)	*n* (%)	*n* (%)
Self-distraction				
	2–5	55 (84.6)	29 (85.3)	26 (83.9)
	6–8	10 (15.4)	5 (14.7)	5 (16.1)
Active coping				
	2–5	44 (67.7)	23 (67.6)	21 (67.7)
	6–8	21 (32.3)	11 (32.4)	10 (32.3)
Denial				
	2–5	59 (93.7)	31 (93.9)	28 (93.3)
	6–8	4 (6.3)	2 (6.1)	2 (6.7)
Missing data		2	1	1
Substance use				
	2–5	58 (89.2)	30 (88.2)	28 (90.3)
	6–8	7 (10.8)	4 (11.8)	3 (9.7)
Use of emotional support				
	2–5	45 (69.2)	20 (58.8)	25 (80.6)
	6–8	20 (30.8)	14 (41.2)	6 (19.4)
Use of instrumental support				
	2–5	43 (66.2)	19 (55.9)	24 (77.4)
	6–8	22 (33.8)	15 (44.1)	7 (22.6)
Behavioral disengagement				
	2–5	55 (84.6)	26 (76.5)	29 (93.5)
	6–8	10 (15.4)	8 (23.5)	2 (6.5)
Venting				
	2–5	57 (87.7)	27 (79.4)	30 (96.8)
	6–8	8 (12.3)	7 (20.6)	1 (3.2)
Positive reframing				
	2–5	40 (61.5)	19 (55.9)	21 (67.7)
	6–8	25 (38.5)	15 (44.1)	10 (32.3)
Planning				
	2–5	44 (68.8)	21 (61.8)	23 (76.7)
	6–8	20 (31.2)	13 (38.2)	7 (23.3)
Missing data		1	0	1
Humor				
	2–5	57 (87.7)	29 (85.3)	28 (90.3)
	6–8	8 (12.3)	5 (14.7)	3 (9.7)
Acceptance				
	2–5	15 (23.1)	6 (17.6)	9 (29.0)
	6–8	50 (76.9)	28 (82.4)	22 (71.0)
Religion				
	2–5	62 (95.4)	33 (97.1)	29 (93.5)
	6–8	3 (4.6)	1 (2.9)	2 (6.5)
Self-blame				
	2–5	60 (93.8)	30 (90.9)	30 (96.8)
	6–8	4 (6.2)	3 (9.1)	1 (3.2)
Missing data		1	1	0

*Abbreviations*: *Brief COPE* Brief Coping Orientation to Problem Experienced Inventory

2–5: Total score for “I haven’t been doing this at all” and “A little bit” in the Brief COPE

6–8: Total score for “A medium amount” and “I’ve been doing this a lot” in the Brief COPE

Numbers are shown as *n* (%)

### Longitudinal relationships between appetite, coping strategies, and change in morale

**Fig. 2:**
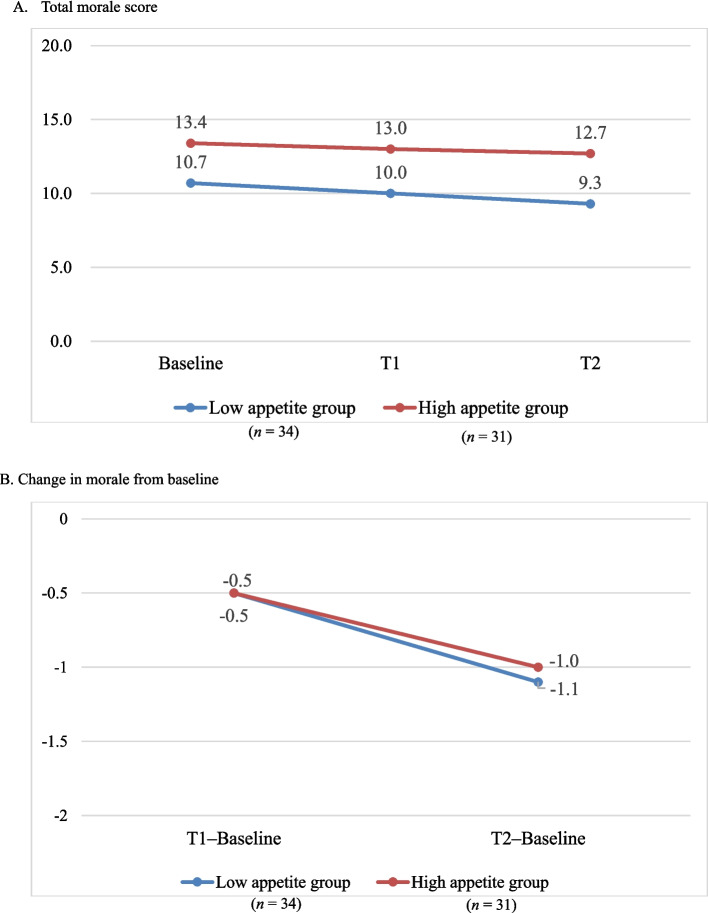
Changes in morale from baseline to T2 in the appetite groups Abbreviations: T1, after one month; T2, after three months

Next, we analyzed the longitudinal relationships between appetite, coping strategies, and changes in morale. The changes in morale from baseline to T2 are shown in Fig. 2 and Table 4, which demonstrate that morale decreased in both groups over the three months. The morale scores of the low-appetite group decreased from 10.7 (intermediate) to the 9-point range (low) after three months, and that of the high-appetite group decreased from 13.4 to 12.7 points (moderate) (Fig. 2). Although the high-appetite group maintained higher scores compared with the low-appetite group, the mixed-effects model (adjusted for age, sex, metastasis, and baseline morale) showed no significant difference in the change in morale between the two groups at three months (intergroup difference: −0.60, 95% confidence interval: −2.01 to 0.82, *p* = 0.40) (Table 4).

**Table 4:** Change in morale from baseline at T2 (adjusted for age, sex, metastasis, and baseline morale)

			Intergroup difference	
	Score level	Estimate (95% CI)	Estimate (95% CI)	*p*-value
SNAQ				
Low-appetite group	-	−1.33 (−2.30 to −0.36)	−0.60 (−2.01 to 0.82)	0.40
High-appetite group	-	−0.73 (−1.69 to 0.23)		
Coping strategies				
Self-distraction	2–5	−1.07 (−1.77 to −0.37)	−0.39 (−2.42 to 1.65)	0.70
	6–8	−0.69 (−2.59 to 1.21)		
Active coping	2–5	−1.17 (−1.96 to −0.39)	−0.52 (−1.96 to 0.92)	0.47
	6–8	−0.65 (−1.85 to 0.54)		
Denial	2–5	−0.92 (−1.57 to −0.27)	−0.24 (−2.56 to 2.08)	0.84
	6–8	−0.68 (−2.91 to 1.55)		
Substance use	2–5	−0.74 (−1.39 to −0.09)	2.72 (0.71 to 4.74)	0.01*
	6–8	−3.46 (−5.36 to −1.56)		
Use of emotional support	2–5	−0.66 (−1.42 to 0.09)	1.28 (−0.14 to 2.69)	0.08
	6–8	−1.94 (−3.13 to −0.75)		
Use of instrumental support	2–5	−1.00 (−1.78 to −0.23)	0.09 (−1.37 to 1.54)	0.90
	6–8	−1.09 (−2.32 to 0.13)		
Behavioral disengagement	2–5	−0.83 (−1.52 to −0.13)	1.54 (−0.44 to 3.52)	0.12
	6–8	−2.37 (−4.22 to −0.52)		
Venting	2–5	−0.95 (−1.64 to −0.26)	0.77 (−1.37 to 2.91)	0.47
	6–8	−1.72 (−3.74 to 0.31)		
Positive reframing	2–5	−1.05 (−1.87 to −0.23)	−0.10 (−1.49 to 1.29)	0.89
	6–8	−0.95 (−2.07 to 0.16)		
Planning	2–5	−1.41 (−2.17 to −0.65)	−1.32 (−2.78 to 0.13)	0.07
	6–8	−0.09 (−1.31 to 1.13)		
Humor	2–5	−1.11 (−1.81 to −0.42)	−0.69 (−2.70 to 1.32)	0.49
	6–8	−0.42 (−2.30 to 1.46)		
Acceptance	2–5	−1.16 (−2.41 to 0.09)	−0.20 (−1.67 to 1.27)	0.79
	6–8	−0.96 (−1.74 to −0.19)		
Religion	2–5	−0.98 (−1.65 to −0.31)	1.16 (−2.12 to 4.43)	0.48
	6–8	−2.14 (−5.34 to 1.07)		
Self-blame	2–5	−0.96 (−1.63 to −0.29)	1.50 (−1.89 to 4.90)	0.38
	6–8	−2.46 (−5.77 to 0.85)		

*Abbreviations*: *Brief COPE* Brief Coping Orientation to Problem Experienced Inventory, *SNAQ* Simplified Nutritional Appetite Questionnaire, *CI* confidence interval

2–5: Total score for “I haven’t been doing this at all” and “A little bit” in the Brief COPE

6–8: Total score for “A medium amount” and “I’ve been doing this a lot” in the Brief COPE

^*^ *p* < 0.05

Regarding the effect of coping strategies on change in morale, the mixed-effects model (Table 4) revealed that, after three months, morale differed significantly through substance use (intergroup difference: 2.74, 95% confidence interval: 0.71 to 4.74, *p* = 0.01). This suggests that individuals who use substances tend to have low morale.

## Discussion

The results showed no significant difference in change in morale after three months between the low- and high-appetite groups. However, coping through substance use led to significant changes in morale. Additionally, appetite (low or high) displayed associations with morale, symptom severity, symptom interference, and frailty at baseline. The lack of significant difference in morale change may be attributed to the fact that morale and appetite declined concurrently in both groups over the three months. As parallel reductions were observed, the results suggest that both groups may have followed similar courses of disease progression, which likely influenced the outcomes equally.

For older adults with gastrointestinal cancer, eating is important for preventing malnutrition, frailty, and mortality. Older adults with cancer following a good diet may be likelier to withstand negative treatment effects [4]. Notably, most participants had a baseline G-8 score of ≤ 14. Although older adults risk becoming malnourished or frail because of cancer treatment [5], the participants had already experienced various symptoms related to cancer and treatment side effects, some of which affected their daily lives and eating habits.

Older adults with gastrointestinal cancer often experience dietary changes because of disease, treatment, and experience-related psychosocial factors [9], and the loss of appetite may lead to reduced social eating and enjoyment of food [3]. For older adults, eating problems have a multifaceted impact, including physical effects, reduced enjoyment of life, and psychological, social, and existential challenges, which can lead to inner instability and decreased morale. In older adults with gastrointestinal cancer, eating problems often trigger psychological distress [3, 9]. However, the lack of significant difference in morale between the appetite groups suggests that coping strategies may be more strongly associated with morale than nutritional status alone. As “acceptance” was the predominant strategy in both groups (76.9%), this shared psychological approach may underlie the similar morale trajectories, appearing to have a stronger connection to morale than the physical differences in appetite. Notably, participants did not often adopt unhealthy or maladaptive strategies such as self-blame, denial, or distraction.

Adaptive coping strategies such as positive reframing, active coping, acceptance, and seeking emotional support can enhance QOL and mood in patients who are aware of their poor prognosis [32]. We presume that both participant groups accepted their circumstances and used planning and positive reframing to cope during difficult situations when their condition worsened and when a new treatment regimen was introduced. Therefore, despite worsening conditions and appetite problems over the three months, participants could cope with disease progression and worsening side effects without their morale being affected.

Substance use was significantly associated with decreased morale over the follow-up period. While the Brief COPE defines “substance use” broadly to encompass alcohol and drugs used for non-medical purposes, given the strict regulations and cultural context in Japan, this finding in our population likely reflects increased alcohol consumption or the maladaptive use of prescribed medications (e.g., sleep aids) rather than the use of prohibited substances. Participants experiencing long-term stress and anxiety due to cancer progression may resort to these substances to manage their distress. Although we reviewed the patients’ history of alcohol consumption and prescribed medications for symptom control, the specific reasons for this substance use remain unclear based on the results of this study. Our results contrast sharply with those of a study on coping among patients with advanced cancer in the United States reporting much lower usage rates (1%) [32]. However, this disparity may stem from cultural reporting biases, such as stigma, which can lead to underreporting in certain patient populations [32]. Regardless of the specific substance, this behavior signals maladaptive coping associated with psychological distress [31] and subsequent morale decline.

Such maladaptive coping strategies are associated with serious psychological issues such as depression and worsening QOL [31], and may ultimately lead to loss of morale. Additionally, the use of emotional support had a negative (albeit insignificant) effect on morale. Some studies have reported a relationship between mood and morale [9, 31] and we believe that the participants were in emotionally distressing situations requiring support because their lives were threatened by cancer recurrence or progression in the long term. This negative trend may reflect that those who frequently seek emotional support are precisely the ones experiencing the most severe distress and decline in morale. Patients with cancer with adequate support can use effective coping strategies (e.g., positive reframing, acceptance, and active coping) to reduce depression and anxiety [33]. Even in situations where cancer progression and aging-related decline in physical function are inevitable, a decline in morale may be prevented by employing positive and adaptive coping strategies to address challenging issues. This highlights the importance of social support from healthcare providers and others as well as the use of available tools.

Research on healthy older adults suggests that increased social support and happiness can help maintain morale [34]. Similarly, self-efficacy and social support positively affect successful aging, even in the presence of health and/or functional changes [10], and eudaimonic well-being can mitigate the negative impact of low subjective health or poor physical health status on QOL [35]. Thus, maintaining the joy of food, effectively utilizing the coping strategies and abilities accumulated over one’s life, and enhancing social support should help preserve and improve older adults’ morale, even when confronted with difficulties such as the progression of gastrointestinal cancer and symptoms and their impact on eating.

### Limitations

Our study has several limitations. First, the setting was a single hospital, which may limit the generalizability of the findings. Second, a formal sample size calculation was not performed. Furthermore, the final sample size was smaller than our initial target despite the extended recruitment period. This limitation was exacerbated by a 16.5% attrition rate due to worsening health or death. Consequently, the reduced statistical power might have limited our ability to detect small differences, which might have contributed to the non-significant results regarding the difference in morale change between appetite groups. To address the potential attrition bias and handle missing data, we employed a mixed-effects model; however, the results should be interpreted with caution, particularly considering the 95% confidence intervals. Third, the heterogeneity of the sample—including different cancer types (upper and lower gastrointestinal cancer) and previous treatments—may have influenced the observed associations. Fourth, the change in coping strategies and morale may require a longer follow-up period to fully assess and confirm any meaningful changes in these variables. Fifth, the use of a self-report questionnaire such as the Brief COPE could lead to potential response biases (under- or overreporting). Finally, this study is a focused investigation into older adults with gastrointestinal cancer; therefore, the observed eating/nutritional issues and age-specific coping strategies may not be generalizable to other cancer types or younger patients.

### Clinical implications

Our findings highlight the importance of addressing the link between eating problems and reduced morale. We propose three specific approaches. First, continued counseling for and monitoring of substance use (including alcohol and medication misuse) may be beneficial, as these behaviors were directly linked to morale decline. Second, for older adults with advanced gastrointestinal cancer, nutritional support should ideally focus on the joy of eating rather than solely on maintaining or managing caloric intake. As appetite loss alone did not predict morale changes, interventions focusing on social eating and personal dietary values might prove more effective. Third, psychological support is recommended to reinforce adaptive “acceptance” while identifying patients actively “seeking support.” These individuals often experience severe distress and may require targeted counseling to prevent depression. Future research should involve multicenter collaborations with control groups of younger patients and those with other cancer types.

## Conclusions

While low baseline appetite was associated with lower morale, it was not significantly associated with changes in morale over three months. Instead, the adoption of substance use as a coping strategy appeared to play a more critical role. Therefore, effective care for older adults with cancer must extend beyond physical symptom management. It is essential to provide comprehensive support that integrates nutritional care focused on the joy of eating—grounded in an understanding of the patient’s values and beliefs—with psychological support that fosters adaptive coping. Such a holistic approach is likely to contribute to enhancing daily enjoyment and morale in this population.

## Supplementary Information

Supplementary Material 1.

Supplementary Material 2.

## Data Availability

The dataset supporting the findings of this study cannot be made publicly available for confidentiality and individual privacy reasons. Data are however available from the corresponding author upon reasonable request.

## Abbreviations

ALB: Serum albumin
CRP: C-reactive protein
BMI: Body mass index
COPE: Coping Orientation to Problems Experienced Inventory
ECOG: Eastern Cooperative Oncology Group
G-8: Geriatric 8 scale
MDASI-J: MD Anderson Symptom Inventory-Japanese Version
NSAIDs: Nonsteroidal anti-inflammatory drugs
PGCMS: Philadelphia Geriatric Center Morale Scale
PGCMS-Re: 17-Item Philadelphia Geriatric Center Morale Scale
QOL: Quality of life
SNAQ: Simplified Nutritional Appetite Questionnaire

## References

[1] Wang S, Zheng R, Li J, Zeng H, Li L, Chen R, et al. Global, regional, and national lifetime risks of developing and dying from gastrointestinal cancers in 185 countries: a population-based systematic analysis of GLOBOCAN. Lancet Gastroenterol Hepatol. 2024;9:229–37. 10.1016/S2468-1253(23)00366-7. 38185129 10.1016/S2468-1253(23)00366-7 PMC10849975

[2] Mislang AR, Di Donato S, Hubbard J, Krishna L, Mottino G, Bozzetti F, et al. Nutritional management of older adults with gastrointestinal cancers: an International Society of Geriatric Oncology (SIOG) review paper. J Geriatr Oncol. 2018;9:382–92. 10.1016/j.jgo.2018.01.003. 29396234 10.1016/j.jgo.2018.01.003

[3] Cope DG. Enhancing mobility and well-being in older adults with cancer. Semin Oncol Nurs. 2024;40:151674. 10.1016/j.soncn.2024.151674. 38965023 10.1016/j.soncn.2024.151674

[4] Doğan Akagündüz DD, Şahin H, Akagündüz B. Malnutrition and related factors in older patients with gastrointestinal cancer receiving chemotherapy. Cureus. 2024;16:e58252. 10.7759/cureus.58252. 38745807 10.7759/cureus.58252 PMC11093618

[5] Qiu J, Xu Y, Xie H, Cai Z, Yang B, Yan Z. An analysis of nutritional risk factors in older adults with gastrointestinal tumours. J Geriatr Oncol. 2023;14:101499. 10.1016/j.jgo.2023.101499. 37120888 10.1016/j.jgo.2023.101499

[6] Hopkinson J. Psychosocial support in cancer cachexia syndrome: the evidence for supported self-management of eating problems during radiotherapy or chemotherapy treatment. Asia Pac J Oncol Nurs. 2018;5:358–68. 10.4103/apjon.apjon_12_18. 30271817 10.4103/apjon.apjon_12_18 PMC6103201

[7] Postma EM, Kok DE, de Graaf CD, Kampman E, Boesveldt S. Chemosensory perception and food preferences in colorectal cancer patients undergoing adjuvant chemotherapy. Clin Nutr ESPEN. 2020;40:242–51. 10.1016/j.clnesp.2020.09.012. 33183544 10.1016/j.clnesp.2020.09.012

[8] Inci H, Inci F, Ersoy S, Karatas F, Adahan D. Self-esteem, metacognition, and coping strategies in cancer patients: a case-control study. J Cancer Res Ther. 2021;17:956–62. 10.4103/jcrt.JCRT_618_19. 34528548 10.4103/jcrt.JCRT_618_19

[9] Yagasaki K, Komatsu H, Hamamoto Y. Rediscovering the joy of eating in older adults with gastrointestinal cancer undergoing treatment: a qualitative study. Cancer Care Res Online. 2022;2:e017. 10.1097/CR9.0000000000000017.

[10] Tovel H, Carmel S. Maintaining successful aging: the role of coping patterns and resources. J Happiness Stud. 2014;15:255–70. 10.1007/s10902-013-9420-4.

[11] Sullivan MD. Maintaining good morale in old age. West J Med. 1997;167:276–84. 9348760 PMC1304544

[12] von Heideken Wågert P, Rönnmark B, Rosendahl E, Lundin-Olsson L, Gustavsson JMC, Nygren B, et al. Morale in the oldest old: the Umeå 85+ study. Age Ageing. 2005;34:249–55. 10.1093/ageing/afi044. 15784647 10.1093/ageing/afi044

[13] Lawton MP. The Philadelphia Geriatric Center Morale Scale: a revision. J Gerontol. 1975;30:85–9. 10.1093/geronj/30.1.85. 1109399 10.1093/geronj/30.1.85

[14] Niklasson J, Näsman M, Nyqvist F, Conradsson M, Olofsson B, Lövheim H, et al. Higher morale is associated with lower risk of depressive disorders five years later among very old people. Arch Gerontol Geriatr. 2017;69:61–8. 10.1016/j.archger.2016.11.008. 27889589 10.1016/j.archger.2016.11.008

[15] Pourtaghi F, Ramezani M, Vashani HB, Hamedi Z, Moghadam ZE. Relationship between depression and social support and morale in the elderly. J Nurs Midwifery Sci. 2019;6:197–203. 10.4103/jnms.jnms_22_19.

[16] Pinar R, Oz H. Validity and reliability of the Philadelphia Geriatric Center Morale Scale among Turkish elderly people. Qual Life Res. 2011;20:9–18. 10.1007/s11136-010-9723-4. 20694856 10.1007/s11136-010-9723-4

[17] Lawton MP. Lawton’s PGC morale scale. 2003. http://www.abramsoncenter.org/PRI/documents/PGC_morale_scale.pdf. Accessed 8 Jan 2025.

[18] Koyano W. An analysis of the revised Philadelphia Geriatric Center Morale Scale. Jpn J Gerontol. 1981;3:83–95.

[19] Ishihara O, Shimonaka Y, Nakazato K, Kawai C, Gondo Y. Stability of the score of PGC morale scale over a five-year period. Jpn J Gerontol. 1999;21:339–45.

[20] Nakatsu N, Sawa R, Misu S, Ueda Y, Ono R. Reliability and validity of the Japanese version of the Simplified Nutritional Appetite Questionnaire in community-dwelling older adults. Geriatr Gerontol Int. 2015;15:1264–9. 10.1111/ggi.12426. 25511474 10.1111/ggi.12426

[21] Wilson MMG, Thomas DR, Rubenstein LZ, Chibnall JT, Anderson S, Baxi A, et al. Appetite assessment: simple appetite questionnaire predicts weight loss in community-dwelling adults and nursing home residents. Am J Clin Nutr. 2005;82:1074–81. 10.1093/ajcn/82.5.1074. 16280441 10.1093/ajcn/82.5.1074

[22] Carver CS, Scheier MF, Weintraub JK. Assessing coping strategies: a theoretically based approach. J Pers Soc Psychol. 1989;56:267–83. Carver CS, Scheier MF, Weintraub JK. Assessing coping strategies: a theoretically based approach. J Pers Soc Psychol. 1989;56:267–83. 10.1037//0022-3514.56.2.267. 2926629 10.1037//0022-3514.56.2.267

[23] Carver CS. You want to measure coping but your protocol’s too long: consider the Brief COPE. Int J Behav Med. 1997;4:92–100. 10.1207/s15327558ijbm0401_6. 16250744 10.1207/s15327558ijbm0401_6

[24] Otsuka Y. The COPE inventory: a theoretically based coping questionnaire. Hiroshima Psychol Res. 2008;8:121–8. 10.15027/26794. Japanese.

[25] NovoPsych. Coping orientation to problems experienced inventory (Brief-COPE). 2025. https://novopsych.com.au/assessments/formulation/brief-cope/. Accessed 25 Mar 2025.

[26] Cleeland CS, Mendoza TR, Wang XS, Chou C, Harle MT, Morrissey M, et al. Assessing symptom distress in cancer patients: the M.D. Anderson Symptom Inventory. Cancer. 2000;89:1634–46. 10.1002/1097-0142(20001001)89:7lt;1634::AID-CNCR29gt;3.0.CO;2-V. 10.1002/1097-0142(20001001)89:7<1634::aid-cncr29>3.0.co;2-v 11013380

[27] Okuyama T, Wang XS, Akechi T, Mendoza TR, Hosaka T, Cleeland CS, et al. Japanese version of the MD Anderson Symptom Inventory: a validation study. J Pain Symptom Manag. 2003;26:1093–104. 10.1016/j.jpainsymman.2003.05.003. 10.1016/j.jpainsymman.2003.05.003 14654261

[28] Bellera CA, Rainfray M, Mathoulin-Pélissier S, Mertens C, Delva F, Fonck M, et al. Screening older cancer patients: first evaluation of the G-8 geriatric screening tool. Ann Oncol. 2012;23:2166–72. 10.1093/annonc/mdr587. 22250183 10.1093/annonc/mdr587

[29] Vellas B, Guigoz Y, Garry PJ, Nourhashemi F, Bennahum D, Lauque S, et al. The Mini Nutritional Assessment (MNA) and its use in grading the nutritional state of elderly patients. Nutrition. 1999;15:116–22. 10.1016/S0899-9007(98)00171-3. 9990575 10.1016/s0899-9007(98)00171-3

[30] Shimaoka H, Yoshida Y, Yamada T, Shimokoube H, Aisu N, Ogawa S, et al. Distribution of Geriatric 8 screening tool scores in elderly and non-elderly patients with cancer. Int J Clin Oncol. 2025;30:457–68. 10.1007/s10147-024-02688-9. 39775670 10.1007/s10147-024-02688-9

[31] Nipp RD, Greer JA, El-Jawahri A, Moran SM, Traeger L, Jacobs JM, et al. Coping and prognostic awareness in patients with advanced cancer. J Clin Oncol. 2017;35:2551–7. 10.1200/JCO.2016.71.3404. 28574777 10.1200/JCO.2016.71.3404 PMC5536163

[32] Dev R, Agosta M, Fellman B, Reddy A, Baldwin S, Arthur J, et al. Coping strategies and associated symptom burden among patients with advanced cancer. Oncologist. 2024;29:166–75. 10.1093/oncolo/oyad253. 37669020 10.1093/oncolo/oyad253 PMC10836315

[33] Zamanian H, Amini-Tehrani M, Jalali Z, Daryaafzoon M, Ala S, Tabrizian S, et al. Perceived social support, coping strategies, anxiety and depression among women with breast cancer: evaluation of a mediation model. Eur J Oncol Nurs. 2021;50:101892. 10.1016/j.ejon.2020.101892. 33583690 10.1016/j.ejon.2020.101892

[34] Ahmed HAAEK, Mohamed BES. Relationship between morality, happiness, and social support among elderly people. Middle East Curr Psychiatry. 2022;29:31. 10.1186/s43045-022-00195-z.

[35] Lopez J, Perez-Rojo G, Noriega C, Sánchez-Cabaco A, Sitges E, Bonete B. Quality-of-life in older adults: its association with emotional distress and psychological wellbeing. BMC Geriatr. 2024;24:815. 10.1186/s12877-024-05401-7. 39385087 10.1186/s12877-024-05401-7 PMC11465940

