# Supplementary material for: Appetite, coping strategies, and morale in older adults with advanced gastrointestinal cancer: a longitudinal observational study
Source: BMC Geriatr. 2026 Apr 21;26:711. doi: 10.1186/s12877-026-07532-5 (PMC13192045; doi:10.1186/s12877-026-07532-5)
Supplement: Supplementary file 1 — Supplementary Material 1. [file 12877_2026_7532_MOESM1_ESM.docx]

**Table S1. Participants’ baseline demographic and clinical characteristics**

| Variables | | | Total  (*n* = 66) | Dropouts  (*n* = 13) |
| --- | --- | --- | --- | --- |
| Sex, *n* (%) | | |  |  |
| Male | | | 35 (53.0) | 6 (46.2) |
| Female | | | 31 (47.0) | 7 (53.8) |
|  | | |  |  |
| Age (years), mean (SD) | | | 76.8 (4.4) | 75.5 (5.0) |
| BMI, mean (SD) | | | 21.3 (3.6) | 20.5 (4.1) |
| CRP (mg/dl), mean (SD), *n* = 65 | | | 0.9 (1.5) | 3.2 (4.9) |
| Albumin (g/dl), mean (SD) | | | 3.6 (0.5) | 3.3 (0.5) |
| Hemoglobin (g/dl), mean (SD) | | | 11.1 (1.7) | 10.9 (1.2) |
| Marital status, *n* (%) | | |  |  |
| Married | | | 59 (93.7) | 11 (84.6) |
| Unmarried | | | 4 (6.3) | 2 (15.4) |
| Missing data | | | 3 |  |
| Living circumstance, *n* (%) | | |  |  |
| Living alone | | | 13 (20.0) | 5 (38.5) |
| Living together | | | 52 (80.0) | 8 (61.5) |
| Missing data | | | 1 |  |
| Alcohol history, *n* (%) | | |  |  |
| No | | | 41 (69.5) | 8 (66.7) |
| Yes | | | 18 (30.5) | 4 (33.3) |
| Cancer site, *n* (%) | | |  |  |
| Colorectal | 26 (39.4) | 4 (30.8) |  |  |
| Hepato-biliary-pancreatic | | 21 (31.8) | 8 (61.5) |  |
| Gastric | | | 13 (19.7) | 1 (7.7) |
| Duodenum | | | 4 (6.1) | 0 (0.0) |
| Small intestine | | | 2 (3.0) | 0 (0.0) |
| Metastasis, *n* (%) | | |  |  |
| Without metastasis | | | 5 (7.6) | 0 (0.0) |
| With metastasis | | | 61 (92.4) | 13 (100.0) |
| History of treatment, *n* (%) | | |  |  |
| Surgery | | | 49 (74.2) | 7 (53.9) |
| Chemotherapy | | | 33 (50.0) | 7 (53.9) |
| Radiotherapy | | | 0 (0.0) | 0 (0.0) |
| ECOG performance status, *n* (%) | | |  |  |
| 0 | | | 30 (45.5) | 1 (7.7) |
| 1 | | | 34 (51.5) | 12 (92.3) |
| 2 | | | 1 (1.5) | 0 (0.0) |
| 3 | | | 1 (1.5) | 0 (0.0) |
| Medication at present, *n* (%) | | |  |  |
| NSAIDs・Acetaminophen | | | 16 (24.2) | 8 (61.5) |
| Opioids | | | 3 (4.6) | 2 (15.4) |
| Sleeping pills | | | 5 (7.6) | 1 (7.7) |
| Antiemetics | | | 17 (25.8) | 4 (30.8) |
| Anamorelin hydrochloride | | | 4 (6.1) | 2 (15.4) |
| Others | | | 52 (78.8) | 13 (100.0) |
| Stoma, *n* (%) | | |  |  |
| With stoma | | | 6 (9.1) | 4 (30.8) |
| None | | | 60 (90.9) | 9 (69.2) |
| SNAQ, mean (SD), *n* = 65 | | | 14.3 (2.1) | 12.4 (2.9) |
| MDASI-J, mean (SD), *n* = 63 | | |  |  |
| Symptom items | | | 1.7 (1.5) | 2.0 (1.8) |
| Interference items | | | 2.1 (1.9) | 3.3 (3.0) |
| G8, mean (SD) | | | 11.7 (2.5) | 10.7 (3.4) |
| PGCMS-Re, mean (SD), *n* = 61 | | | 12.0 (3.0) | 12.7 (2.1) |

Abbreviations: BMI, Body mass index; CRP, C-reactive protein; ECOG, Eastern Cooperative Oncology Group; G8, Geriatric 8; MDASI-J, MD Anderson Symptom Inventory-Japanese Version; NSAIDs, Nonsteroidal anti-inflammatory drugs; Small intestine, small intestine including the cecum; SNAQ, Simplified Nutritional Appetite Questionnaire; PGCMS-Re, Revised Philadelphia Geriatric Center Morale Scale.

Numbers are presented as *n* (%) or means (SD, standard deviation).
