# Supplementary material for: Appetite, coping strategies, and morale in older adults with advanced gastrointestinal cancer: a longitudinal observational study
Source: BMC Geriatr. 2026 Apr 21;26:711. doi: 10.1186/s12877-026-07532-5 (PMC13192045; doi:10.1186/s12877-026-07532-5)
Supplement: Supplementary file 2 — Supplementary Material 2. [file 12877_2026_7532_MOESM2_ESM.docx]

**Table S2. Participants’ coping strategies**

| Variables | Score  level | Total  (*n* = 65) | Dropouts  (*n* = 13) |
| --- | --- | --- | --- |
|  |  | *n* (%) | *n* (%) |
| Self-distraction |  |  |  |
|  | 2–5 | 55 (84.6) | 8 (61.5) |
|  | 6–8 | 10 (15.4) | 5 (38.5) |
| Active coping |  |  |  |
|  | 2–5 | 44 (67.7) | 9 (69.2) |
|  | 6–8 | 21 (32.3) | 4 (30.8) |
| Denial |  |  |  |
|  | 2–5 | 59 (93.7) | 11 (84.6) |
|  | 6–8 | 4 (6.3) | 2 (15.4) |
| Missing data | | 2 | 0 |
| Substance use |  |  |  |
|  | 2–5 | 58 (89.2) | 11 (84.6) |
|  | 6–8 | 7 (10.8) | 2 (15.4) |
| Use of emotional support |  |  |  |
|  | 2–5 | 45 (69.2) | 6 (46.2) |
|  | 6–8 | 20 (30.8) | 7 (53.8) |
| Use of instrumental support |  |  |  |
|  | 2–5 | 43 (66.2) | 9 (69.2) |
|  | 6–8 | 22 (33.8) | 4 (30.8) |
| Behavioral disengagement |  |  |  |
|  | 2–5 | 55 (84.6) | 11 (84.6) |
|  | 6–8 | 10 (15.4) | 2 (15.4) |
| Venting |  |  |  |
|  | 2–5 | 57 (87.7) | 12 (92.3) |
|  | 6–8 | 8 (12.3) | 1 (7.7) |
| Positive reframing |  |  |  |
|  | 2–5 | 40 (61.5) | 8 (61.5) |
|  | 6–8 | 25 (38.5) | 5 (38.5) |
| Planning |  |  |  |
|  | 2–5 | 44 (68.8) | 10 (76.9) |
|  | 6–8 | 20 (31.2) | 3 (23.1) |
| Missing data | | 1 | 0 |
| Humor |  |  |  |
|  | 2–5 | 57 (87.7) | 12 (92.3) |
|  | 6–8 | 8 (12.3) | 1 (7.7) |
| Acceptance |  |  |  |
|  | 2–5 | 15 (23.1) | 6 (46.2) |
|  | 6–8 | 50 (76.9) | 7 (53.8) |
| Religion |  |  |  |
|  | 2–5 | 62 (95.4) | 11 (84.6) |
|  | 6–8 | 3 (4.6) | 2 (15.4) |
| Self-blame |  |  |  |
|  | 2–5 | 60 (93.8) | 11 (84.6) |
|  | 6–8 | 4 (6.2) | 2 (15.4) |
| Missing data | | 1 | 0 |

Abbreviations: Brief COPE, Brief Coping Orientation to Problems Experienced Inventory.

2–5: Total score for “I haven’t been doing this at all” and “A little bit” in the Brief COPE.

6–8: Total score for “A medium amount” and “I’ve been doing this a lot” in the Brief COPE.

Numbers are shown in *n* (%).
